# Preoperative ejection fraction as a predictor of survival after coronary artery bypass grafting: comparison with a matched general population

**DOI:** 10.1186/1749-8090-5-29

**Published:** 2010-04-23

**Authors:** Mohamed A Soliman Hamad, Albert HM van Straten, Jacques PAM Schönberger, Joost F ter Woorst, Andre M de Wolf, Elisabeth J Martens, André AJ van Zundert

**Affiliations:** 1 Department of Cardio-Thoracic Surgery, Catharina Hospital, Eindhoven, The Netherlands; 2 Department of Anesthesiology, The Feinberg School of Medicine, Northwestern University, Chicago, Illinois, USA; 3 Department of Education and Research, Catharina Hospital, Eindhoven, the Netherlands; 4 Center of Research on Psychology in Somatic diseases, Department of Medical Psychology, Tilburg University, the Netherlands; 5 Department of Anesthesiology, Catharina Hospital - Brabant Medical School, Eindhoven, the Netherlands; 6 University Hospital Ghent, Ghent, Belgium

## Abstract

**Background:**

Preoperative left ventricular dysfunction is an established risk factor for early and late mortality after revascularization. This retrospective analysis demonstrates the effects of preoperative ejection fraction on the short-term and long-term survival of patients after coronary artery bypass grafting.

**Methods:**

Early and late mortality were determined retrospectively in 10 626 consecutive patients who underwent isolated coronary bypass between January 1998 and December 2007. The subjects were divided into 3 groups according to their preoperative ejection fraction. Expected survival was estimated by comparison with a general Dutch population group described in the database of the Dutch Central Bureau for Statistics. For each of our groups with a known preoperative ejection fraction, a general Dutch population group was matched for age, sex, and year of operation.

**Results and Discussion:**

One hundred twenty-two patients were lost to follow-up. In 219 patients, the preoperative ejection fraction could not be retrieved. In the remaining patients (n = 10 285), the results of multivariate logistic regression and Cox regression analysis identified the ejection fraction as a predictor of early and late mortality. When we compared long-term survival and expected survival, we found a relatively poorer outcome in all subjects with an ejection fraction of < 50%. In subjects with a preoperative ejection fraction of > 50%, long-term survival exceeded expected survival.

**Conclusions:**

The severity of left ventricular dysfunction was associated with poor survival. Compared with the survival of the matched general population, our coronary bypass patients had a worse outcome only if their preoperative ejection fraction was < 50%.

## Introduction

Despite improvement in medical therapies and surgical techniques, the management of patients with coronary artery disease and a low ejection fraction (EF) remains challenging. In patients with a low EF, coronary artery bypass grafting (CABG) has been shown to be superior to medical therapy alone, to produce a statistically significant clinical improvement, and to improve long-term survival [1-5]. In such patients, however, CABG is associated with higher postoperative morbidity and mortality rates than those in patients whose left ventricular function is within normal limits [5,6]. In an earlier investigation [7], we showed that superior long-term results after CABG occurred in a group of patients with a low EF (< 40%) who were prospectively studied. However, most such reports are limited by inadequate sample size. In this study of patients who underwent CABG, we correlated risk factors and outcomes with preoperative EF and compared the long-term survival of our subjects with that of matched cohorts from the general population of The Netherlands.

## Methods

This retrospective study consisted of 10 626 patients who underwent isolated CABG performed in the Department of Cardiothoracic Surgery at Catharina Hospital in Eindhoven, The Netherlands, between January 1998 and December 2007. After excluding 122 patients who were lost to follow-up and 219 patients whose the preoperative EF was not retrieved, 10285 patients were evaluated. The study was performed after permission from the local medical ethics committee had been received.

### Preoperative EF

The global EF was determined with 1 or both of following methods: calculation with 2-dimensional echocardiography via the biplane apical method and the modified Simpson's rule [8], and/or ventriculographic evaluation performed by an independent surgeon and an independent cardiologist. The patients were divided into 3 groups as follows: group 1, EF > 50% (n = 8204); group 2, EF = 35% to 50% (n = 1717); group 3, EF < 35% (n = 364).

### Operative techniques

All patients received short-acting anesthetic drugs to facilitate early extubation. Extracorporeal circulation was performed via a normothermic nonpulsatile flow. Cold crystalloid cardioplegia ("St. Thomas solution") or warm-blood cardioplegia was used according to the surgeon's preference to induce and maintain cardioplegic arrest.

### Follow-Up

Follow-up data on mortality were gathered from the databases of health insurance companies, general practitioners, and (if necessary) the governmental authorities. Early mortality was defined as death that occurred from any cause within the first 30 postoperative days, and late mortality was defined as death that occurred more than 30 days after surgery, regardless of cause. For calculating survival of a general population cohort, data were obtained from the Dutch Central Bureau for Statistics (CBS). This is the database registering information about all citizens living in the Netherlands. Every year, a report from the CBS is available online about mortality within the normal population stratified by age and sex. We have matched each group in our study with the general population according to age and sex. Because the incidence of mortality within the general population varies per year, the matching was also done to compare the survival of each group with the survival of the general population for the same year when the studied patients were operated. We considered the survival of the matched general population cohort to represent the expected survival of the patient group.

### Statistical analyses

Discrete variables, which were compared by means of the chi-squared test, are presented as numbers and percentages. Continuous variables were compared by means of the *t *test and analysis of variance and are presented as the mean ± standard deviation. Univariate and multivariate logistic regression analyses were performed to investigate the impact of biomedical variables on early mortality. Univariate analyses were used to test potentially confounding effects of biomedical and demographic factors on outcome. The Cox proportional hazard regression analysis was performed to evaluate late mortality. If the *P *value decreased to < .05, then confounding variables were included in the multivariate logistic and Cox regression analyses. Long-term survival was depicted with the Kaplan-Meier method. For comparisons of long-term survival, we used log-rank statistics. "Time zero" was used to designate the time of CABG. The results of timetable analyses were used to describe 5-year and 10-year survival, and comparisons were made with the Wilcoxon test. For all tests, a *P *value of < .05 indicated statistical significance. Hazard ratios are reported with 95% confidence intervals. All statistical analyses were performed with SPSS software (Statistical Product and Service Solutions, version 15.0, SSPS Inc, Chicago, Illinois).

## Results

The minimum follow-up interval for surviving patients was 60 days. The mean follow-up period was 1696 ± 1026 days (range, zero to 3708 days; day zero represented operative death).

The baseline characteristics of patients in the various EF groups are represented in Table 1. Table 2 shows the operative details of patients in those EF groups. Patients with a low EF (groups 2 and 3) had a longer extracorporeal circulation time than did the other subjects and were more likely to require perioperative intra-aortic balloon pump support than were patients whose EF was within normal limits. There were also fewer off-pump operations in patients with a low EF.

**Table 1:** Preoperative characteristics of the study subjects*.

Variables	Group 1 (EF > 50%) (n = 8204)	Group 2 (EF = 35% -50%) (n = 1717)	Group 3 (EF < 35%) (n = 364)	*P *Value
Age (y) (mean ± SD)	64.5 ± 9.5	65.0 ± 9.7	65.6 ± 8.9	0.014
Male sex (%)	6254 (76.2)	1382 (80.5)	297 (81.6)	< 0.0001
NYHA class III or IV (%)	436 (5.3)	142 (8.3)	53 (14.6)	< 0.0001
Angina class (mean ± SD)	2.7 ± 1.2	2.6 ± .3	2.3 ± 1.5	0.012
Hypertension (%)	3554 (43.3)	649 (37.8)	127 (34.9)	< 0.0001
COPD (%)	987 (12.0)	258 (15.0)	58 (15.9)	< .0001
Diabetes (%)	1692 (20.6)	413 (24.1)	96 (26.4)	< 0.0001
1 Prior MI (%)	2635 (32.3)	1016 (59.3)	204 (56.0)	< 0.0001
2 Prior MIs (%)	280 (3.4)	167 (9.7)	55 (15.1)	< 0.0001
> 2 Prior MIs (%)	25 (0.3)	15 (0.9)	6 (1.6)	< 0.0001
CrCl < 60 mL/min (%)	2168 (27.4)	568 (34.4)	152 (44.4)	< 0.0001
PVD (%)	908 (11.1)	239 (13.9)	51 (14.0)	0.002
Emergency (%)	237 (2.9)	71 (4.1)	33 (9.1)	< 0.0001
Prior cardiac surgery (%)	384 (4.7)	154 (9.0)	39 (10.7)	< 0.0001

EF = Ejection fraction, NYHA = New York Heart Association, COPD = chronic obstructive pulmonary disease, MI = myocardial infarction, CrCl = creatinine clearance, PVD = peripheral vascular disease.

*Data are represented as number (percent) or as the mean ± SD.

**Table 2:** Operative details of the study subjects*.

Variables	Group 1 (EF > 50%) (n = 8204)	Group 2 (EF = 35% -50%) (n = 1717)	Group 3 (EF < 35%) (n = 364)	*P *Value
Off-pump (%)	780 (9.5)	101 (5.9)	21 (5.8)	< 0.0001
IMA (%)	7378 (89.9)	1494 (87.0)	277 (76.1)	< 0.0001
No. anastomoses (mean ± SD)	3.42 ± 1.1	3.5 ± 1.1	3.62 ± 1.1	0.295
Cardioplegia:				
Crystalloid (%)	2432 (36.4)	479 (38.1)	118 (42.0)	< 0.001
Blood (%)	3299 (49.35)	652 (51.9)	133 (47.3)	< 0.001
ECC time (min) (mean ± SD)	56.5 ± 32.7	61.9 ± 31.0	68.0 ± 35.8	< 0.001
Re-exploration (%)	428 (5.2)	119 (6.9)	13(3.6)	0.009
Perioperative MI (%)	240 (2.9)	49 (2.9)	12 (3.3)	0.775
IABP (%)	121 (1.5)	56 (3.3)	30 (8.2)	< 0.0001

EF = Ejection fraction, IMA = internal mammary artery, ECC = extracorporeal circulation, MI = myocardial infarction, IABP = intra-aortic balloon pump support.

*Data are represented as the number (%) or mean ± SD.

Early and late mortality were statistically significantly higher in patients with a lower EF (Table 3). Risk factors for early mortality identified by univariate and multivariate logistic regression analyses are shown in Table 4. Univariate logistic regression analysis identified preoperative EF as a risk factor for early mortality. However, the hazard ratio was higher in patients with an EF of < 35% than in those with an EF of 35% to 50%. Other risk factors identified by univariate analysis included age, New York Heart Association class, diabetes, chronic obstructive pulmonary disease (COPD), peripheral vascular disease (PVD), anemia, renal dysfunction, prior cardiac surgery, and emergency operation. Perioperative complications such as myocardial infarction, the need for intra-aortic balloon pump support, and re-exploration were also identified as risk factors for early mortality.

**Table 3:** Early and late mortality according to preoperative ejection fraction.

Variables	Group 1 (EF > 50%) (n = 8204)	Group 2 (EF = 35% -50%) (n = 1717)	Group 3 (EF < 35%) (n = 364)	*P *Value
Early mortality (%)	129 (1.6)	63 (3.7)	38 (10.5)	< .0001
Late mortality (%)	742 (9.1)	296 (17.4)	81 (22.4)	< .0001

EF = Ejection fraction.

**Table 4:** Univariate and multivariate logistic regression analyses of risk factors for early mortality in the study subjects†.

Risk factors	OR early mortality Univariate analysis	P value	OR early mortality Multivariate analysis	P value
EF 35% - 50%	2.623 (1.923 -3.578)	< .0001	1.9 (1.335 -12.693)	< .0001
EF < 35%	7.592 (5.143 -11.207)	< .0001	4.206 (2.6 -6.805)	< .0001
Age (y)*	1.08 (1.062 -1.098)	< .0001	1.031 (1.004 -1.059)	.026
Male sex	0.802 (0.601 -1.070)	.071		
NYHA class	1.338 (1.122 -1.595)	.001	1.168 (0.908 -1.503)	.227
Angina class	1.002 (0.951 -1.055)	.277		
Hypertension	0.929 (0.716 -1.207)	.662		
COPD	1.966 (1.438 -2.687)	< .0001	1.479 (0.943 -2.319)	.089
Preoperative Hb level	0.692 (0.634 -0.755)	< .0001	0.883 (0.779 -1.001	.051
Diabetes	1.524 (1.148 -2.023)	.004	1.743 (1.195 -2.543)	.004
Preoperative CrCl	0.965 (0.962 -0.968)	< .0001	0.978 (0.967 -0.988)	< .0001
PVD	1.633 (1.164 -2.290)	.005	1.441 (991 -2.277)	.118
Prior cardiac surgery	4.542 (3.304 -6.244)	< .0001	3.064 (1.847 -5.083)	< .0001
No. of anastomoses	0.903 (0.801-1.011)	.077		
Off-pump	0.631 (0.366 -1.087)	.097		
Use of IMA	0.231 (0.176 -0.303)	< .001		
Cardioplegia	1.312 (0.995 -1.731)	.054		
Emergency	6.550 (4.722 -9.087)	< .0001	3.307 (1.597 -6.846)	.001
Perioperative MI	5.938 (4.053 -8.699)	< .0001		
Re-exploration	5.810 (4.261-7.922)	< .0001		
IABP	13.974 (9.916 -19.691)	< .0001		

OR = Odds ratio, EF = ejection fraction, NYHA = New York Heart Association, COPD = chronic obstructive pulmonary disease, Hb = hemoglobin, CrCl = creatinine clearance, PVD = peripheral vascular disease, IMA = internal mammary artery, MI = myocardial infarction, IABP = intra-aortic balloon pump support.

† Only preoperative variables which are significant in the univariate analysis were entered into the multivariate analysis

*Entered as a continuous variable.

All preoperative risk factors identified by univariate analysis were entered in the multivariate logistic regression model. A low EF proved to be an independent risk factor for early mortality. Other factors were age, diabetes, COPD, renal dysfunction, prior cardiac surgery, and emergency operation.

The results of Cox regression analysis to identify risk factors for late mortality are shown in Table 5. Univariate analysis identified preoperative EF as a risk factor for late mortality. Other significant risk factors were age, sex, New York Heart Association class, hypertension, anemia, COPD, diabetes, renal dysfunction, PVD, and prior cardiac surgery. When those factors were entered into the multivariate analysis, a low EF proved to be an independent risk factor for late mortality. Other statistically significant factors were age, sex, New York Heart Association class, diabetes, COPD, renal dysfunction, anemia, PVD, and prior cardiac surgery.

**Table 5:** Univariate and multivariate Cox regression analyses of risk factors for late mortality†.

Risk factor	HR late mortality Univariate analysis	P value	HR late mortality Multivariate analysis	P value
EF 35% -50%	1.866 (1.614 -2.157)	< .0001	1.562 (1.339 -1.822)	< .0001
EF < 35%	2.859 (2.231-3.665)	< .0001	1.051 (0.924 -1.196)	< .0001
Age (y)*	1.094 (1.086 -1.103)	< .0001	1.067 (1.053 -1.081)	< .0001
Male sex	0.835 (0.726 -0.961)	.012	1.629 (1.346 -1.97)	< .0001
NYHA class	1.201 (1.094 -1.318)	< .0001	1.501 (1.267 -1.779)	< .0001
Angina class	1.002 (0.951-1.055)	.95		
Hypertension	1.223 (1.079 -1386)	.002	1.137 (0.971 -1.33)	.11
COPD	1.778 (1.523 -2.077)	< .0001	1.473 (1.211 -1.792)	< .0001
Diabetes	1.733 (1.512 -1.985)	< .0001	1.526 (1.287 -1.809)	< .0001
Preoperative CrCl	0.965 (0962 -0.968)	< .0001	0.986 (0.981 -0.992)	< .0001
PVD	2.307 (1.978 -2.690)	< .0001	1.699 (1.397 -2.066)	< .0001
Preoperative Hb	0.743 (0.711 -0.777)	< .0001	0.867 (0.816 -0.922)	< .0001
Prior cardiac surgery	1.536 (1.248 -1.891)	< .0001	1.143 (0.851-1.536)	.374
Emergency	1.268 (0.957 -1.681)	.099		
No. of anastomoses	1.089 (1.032 -1.148)	.002		
Use of IMA	0.544 (0.465 -0.637)	< .0001		
Off-pump	0.76 (0.58 -0.996)	.046		
Perioperative MI	1.801 (1.331-2.437)	< .0001		
Re-exploration	1.673 (1.344 -2.082)	< .0001		
IABP	1.903 (1.364 -2.655)	< .0001		

HR = Hazard ratio, EF = ejection fraction, NYHA = New York Heart Association, COPD = chronic obstructive pulmonary disease, CrCl = creatinine clearance, PVD = peripheral vascular disease, Hb = hemoglobin, IMA = internal mammary artery, MI = myocardial infarction, IABP = intra-aortic balloon pump support.

† Only preoperative variables which are significant in the univariate analysis were entered into the multivariate analysis

*Entered as a continuous variable.

**Figure 1:**
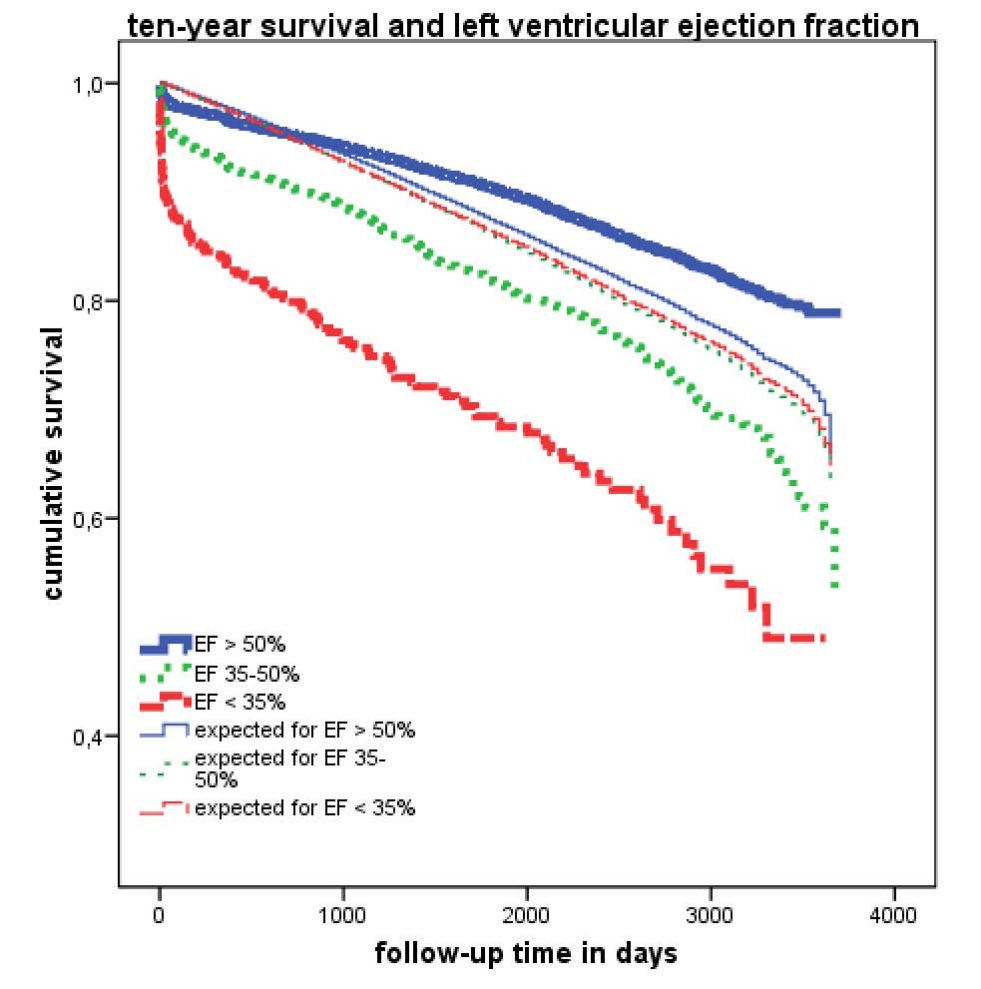
**Kaplan-Meier curve of study groups and their expected survival**.

Figure 1 shows long-term survival stratified by preoperative EF. The log-rank test yielded a *P *value of < .0001, which indicates statistically significant differences in long-term survival among all groups. Patients in group 1 (EF > 50%) had greater long-term survival than that expected (*P *< .0001). However, the long-term survival of patients in both group 2 (EF = 35-50%) and group 3 (EF < 35%) was worse than the expected survival (*P *< .0001; log-rank test). One-year, 5-year and 10-year survival differed among patient groups (Wilcoxon test *P *value < .0001) (Table 6).

**Table 6:** Survival rates (%) for 1, 5, and 10 years, stratified by preoperative ejection fraction (EF).

	1-year	5-year	10-year
Group 1 (EF > 50%)	95.1 ± 0.2	87.9 ± 0.4	78.6 ± 0.9
Group 2 (EF = 35-50%)	90.0 ± 0.8	78.9 ± 0.12	50.7 ± 6.8
Group 3 (EF < 35%)	79.0 ± 2.2	64.8 ± 2.9	44.7 ± 6.5

## Discussion

The main finding of this study was that preoperative EF is a statistically significant predictor for higher rates of early and late mortality after CABG. Patients with a low EF had a worse survival than did patients whose EF was within normal limits. Revascularization in patients with a low EF has been reported by several authors to be superior to medical therapy. Alderman and colleagues [1] showed that patients with an EF of ≤ 35% who were treated with medical management had a 43% 5-year survival rate as opposed to a 63% 5-year survival rate in the surgically treated patients. Although CABG enables longer survival and a better quality of life than does medical therapy, the postsurgical outcomes of patients with a low EF have been shown to be considerably worse than those in patients with a high EF [3,6].

A low EF has been shown to be an independent risk factor for high operative mortality [9,10]. In our study, we noted that the early mortality rate in patients with an EF of < 35% was more than 6 times higher than that in patients with an EF of > 50% (10.5% vs 1.6%). This finding supports the results of other studies on the initial effect of isolated CABG on mortality in patients with a low EF. Di Carli and colleagues [4] reported a 9.3% 30-day mortality rate in patients with an EF of < 40%. Christakis and colleagues [6] demonstrated a 9.8% operative mortality rate in patients with an EF of < 20%, and a study by Carr and colleagues [11] demonstrated an 11% perioperative mortality rate in patients with an EF between 10% and 20%. However, more recent reports have shown lower operative mortality rates. In a review of the New York State database [12], the early mortality rate of patients with an EF of ≤ 20% was 4.6%. Another report showed an in-hospital mortality rate of 4% in patients with an EF of < 30% [13]. In an earlier report, we found approximately the same in-hospital mortality rate (4%) in 75 prospectively studied patients with an EF of < 40% [14]. The decline of those mortality rates over time showed a statistically significant improvement from the double-digit rates reported in the 1980s. We suggest that improvements in cardiac anesthesia, perioperative care, surgical techniques, emergency cardiac care, and postoperative management contribute significantly to more encouraging outcomes.

Patients with impaired left ventricular function who undergo CABG are a distinctive group of patients. Their risk factors that increase the postoperative mortality rate may not be similar to risk factors usually found in patients whose EF is within normal limits. Christakis and colleagues [6] observed that the urgency of surgery was the only independent predictor of operative mortality in patients with an EF of < 20% who underwent CABG. Other authors [15] have reported that an age of > 70 years was the only independent predictor of in-hospital mortality in patients with an EF of ≤ 30% who underwent CABG. Hausmann and colleagues [16] noted that increased left ventricular end diastolic pressures, decreased cardiac index, and New York Heart Association class were univariate predictors of operative mortality in patients with an EF of < 30%. Argenziano and colleagues [17] found that reoperation and congestive heart failure were predictors of perioperative mortality in patients with an EF of ≤ 35%. In our study, patients with a low EF had a higher incidence of preoperative comorbid conditions such as diabetes, New York Heart Association class III or IV, COPD, renal dysfunction, PVD, and/or reoperation than did those with normal EF. Those factors may have contributed to the higher incidence of early mortality in patients with low EF. Using multivariate logistic regression analysis, we found age, New York Heart Association class, renal dysfunction, COPD, diabetes, reoperation, and emergency operation to be statistically significant predictors of in-hospital mortality.

The results of our study confirmed that patients with a lower EF have a poorer long-term outcome than do patients whose EF is within normal limits. We found that in patients with an EF of < 35%, the 5-year survival rate was 64.8%, and the 10-year survival rate was 44.7%. Those statistics compare favorably with the results of medical treatment, even in the current era of aggressive use of angiotensin-converting enzyme inhibitors and other medications for congestive heart failure [18]. In some studies, complete revascularization of the ischemic myocardium had a major impact on long-term survival, even when viability was not consistently documented. Shapira and colleagues [19] noted a 5-year survival of 76% in patients with an EF of < 30% who underwent CABG. Similar results were reported by other investigators [9,20-23]. The number of studies addressing 10-year survival in such patients, however, is limited. In a study by Shah and colleagues [20], the 5-year survival rate in patients with an EF of < 35% was 55%, and the 10-year survival rate was 23.9%. In a recent study of patients with an EF of ≤ 30, approximately 80% were alive 5 years after surgery, and 45% were alive 10 years after surgery [24]. A 20-year survival study by Weintraub and colleagues showed that a low EF independently predicted poor long-term survival after CABG, although the subjects experienced good relief from angina [25].

Like other authors [20,21], we observed that age and male sex are independent predictors of long-term outcome in patients undergoing CABG. Other important predictors were New York Heart Association class, COPD, anemia, renal dysfunction, diabetes, and PVD. Bouchart and colleagues [10] identified the following statistically significant predictors of long-term survival after CABG in patients with an EF of ≤ 20%: a chief complaint of only pain, unstable angina, and a Canadian and New York Heart Association class lower than IV.

Case selection has been shown to be an important factor in achieving a favorable outcome after CABG in patients with a low EF [24]. Our study included patients without preoperative viability test results and those with a ventricular aneurysm or associated mild or moderate mitral regurgitation. Di Carli and colleagues [4] showed that in patients evaluated with positron emission tomography, those who had an EF of < 40% and a viable myocardium had a better 4-year survival rate than did patients without evidence of a viable myocardium.

A rather unique feature of our study is that we compared the survival of our patients with that of a cohort of the general Dutch population matched for age, sex, and year of operation. Over the years, variation in life expectancy and mortality rates of the Dutch population has been well documented by the Dutch Central Bureau for Statistics. We used data from the Central Bureau for Statistics to compare survival of our patients with the survival of general population cohorts matched for age and sex (expected survival). We found that patients with a low EF had worse long-term survival than that their matched cohort of the Dutch citizens. Patients whose EF was within normal limits had better long-term survival than that in the matched cohort of the general Dutch population. Although that information does not guide surgical decision making, it may be relevant for patients with regard to their long-term prognosis. Nevertheless, those findings must be interpreted with caution, because the Dutch Central Bureau for Statistics database includes data from the entire Dutch population. As a result, data from the patients described in this study as well as data from patients treated in other cardiac surgery centers are included. In patients who underwent CABG, the protection provided by revascularization, the postoperative medical therapy administered to treat hypertension and hypercholesterolemia, and the use of antiplatelet therapy may increase the bias. In addition, patients who are scheduled to undergo CABG receive preoperative screening for, and treatment of underlying diseases that may contraindicate surgery. Perhaps for those reasons, survival in patients whose EF was within normal limits was longer than the expected survival in the matched cohort of the *normal *general population.

### Limitations of the study

Like most similar reports, our study was based on the retrospective evaluation of patient charts. To prove the usefulness of a surgical procedure, a study must be prospective, controlled, and randomized. However, we suggest that the relatively large number of patients in our report justifies our conclusions. The primary endpoint of the study was all-cause mortality. We were not able to retrieve the cause of death in both groups which could be equally important. Information about the quality of life of the surviving patients, their eventual symptoms, and their incidence of rehospitalization; residual mitral regurgitation; the recurrence of congestive heart failure; and other possible complications is lacking. We recommend caution in interpreting the results of the comparison with the general population. The Central Bureau for Statistics database includes the total Dutch population. Therefore, data of the patients described in this study and of those treated at other Dutch cardiac surgery centers are also included in the CBS databse. Because of this, the magnitude of differences between groups tends to be lessened. The annual number of patients undergoing CABG in the Netherlands is small, (10 000 patients), compared to the total number of the general population, limiting the effect of this inaccuracy. Clinical information including data about the EF is missing in the general population group. However, the results of our study can help in informing patients with normal preoperative EF that their prognosis after CABG is favourable.

## Conclusions

This study confirmed that a low EF is a predictive risk factor for early and late mortality after CABG. Patients whose EF was within normal limits (ie, > 50%) had better long-term survival than that in a matched cohort of the general Dutch population, but patients with a low EF (ie, < 50%) had a worse long-term survival than that in their respective matched cohort.

## Authors' contributions

**MSH**: Participated in the design of the study, writing the manuscript and performed the revisions. **AvS**: participated in the design of the study, performing the statistical analysis, and writing the manuscript. **JS**: participated in writing and revising the manuscript. **JtW**: participated in writing the manuscript. **AdW**: participated in writing the manuscript. **EM**: participated in the statistical analysis. **AvZ**: participated in writing and revising the manuscript. All authors read and approved the final manuscript.

## Competing interests

The authors declare that they have no competing interests.
